# Study of Microbial Sulfur Metabolism in a Near Real-Time Pathway through Confocal Raman Quantitative 3D Imaging

**DOI:** 10.1128/spectrum.03678-22

**Published:** 2023-02-21

**Authors:** Wanying He, Ruining Cai, Shichuan Xi, Ziyu Yin, Zengfeng Du, Zhendong Luan, Chaomin Sun, Xin Zhang

**Affiliations:** a CAS Key Laboratory of Marine Geology and Environment & Center of Deep Sea Research, Institute of Oceanology, Chinese Academy of Sciences, Qingdao, China; b Laboratory for Marine Geology, Pilot Laboratory for Marine Science and Technology, Qingdao, China; c College of Earth Science, University of Chinese Academy of Sciences, Beijing, China; d CAS Key Laboratory of Experimental Marine Biology & Center of Deep Sea Research, Institute of Oceanology, Chinese Academy of Sciences, Qingdao, China; e Laboratory for Marine Biology and Biotechnology, Pilot National Laboratory for Marine Science and Technology, Qingdao, China; Georgia Institute of Technology

**Keywords:** deep-sea bacteria, sulfur metabolism, confocal Raman microscopy, *in situ*, near real time

## Abstract

As microbial sulfur metabolism significantly contributes to the formation and cycling of deep-sea sulfur, studying their sulfur metabolism is important for understanding the deep-sea sulfur cycle. However, conventional methods are limited in near real-time studies of bacterial metabolism. Recently, Raman spectroscopy has been widely used in studies on biological metabolism due to its low-cost, rapid, label-free, and nondestructive features, providing us with new approaches to solve the above limitation. Here, we used the confocal Raman quantitative 3D imaging method to nondestructively detect the growth and metabolism of Erythrobacter flavus 21-3 in the long term and near real time, which possessed a pathway mediating the formation of elemental sulfur in the deep sea, but the dynamic process was unknown. In this study, its dynamic sulfur metabolism was visualized and quantitatively assessed in near real time using 3D imaging and related calculations. Based on 3D imaging, the growth and metabolism of microbial colonies growing under both hyperoxic and hypoxic conditions were quantified by volume calculation and ratio analysis. Additionally, unprecedented details of growth and metabolism were uncovered by this method. Due to this successful application, this method is potentially significant for analyzing the *in situ* biological processes of microorganisms in the future.

**IMPORTANCE** Microorganisms contribute significantly to the formation of deep-sea elemental sulfur, so studies on their growth and dynamic sulfur metabolism are important to understand the deep-sea sulfur cycle. However, near real-time *in situ* nondestructive metabolic studies of microorganisms remain a great challenge due to the limitations of existing methods. We thus used an imaging-related workflow by confocal Raman microscopy. More detailed descriptions of the sulfur metabolism of *E. flavus* 21-3 were disclosed, which perfectly complemented previous research results. Therefore, this method is potentially significant for analyzing the *in-situ* biological processes of microorganisms in the future. To our knowledge, this is the first label-free and nondestructive *in situ* technique that can provide temporally persistent 3D visualization and quantitative information about bacteria.

## INTRODUCTION

Elemental sulfur is widely distributed on the deep-sea floor and is formed by the oxidation of reduced sulfur compounds, which is mediated chemically or microbially (1–4). Microorganisms are important contributors to the formation and cycling of deep-sea sulfur as the main driver (5). Moreover, generating and utilizing elemental sulfur are important survival strategies for bacteria participating in the deep-sea sulfur cycle (6). Therefore, studies on their sulfur metabolism are essential to understand the deep-sea sulfur cycle. However, studies are still limited due to the lack of sampling and culture conditions (7, 8) and methods for monitoring the formation of elemental sulfur.

Bacteria producing elemental sulfur isolated from the environment except the deep sea have been studied for a long time. They belong to *Acidithiobacillia*, *Alpha-*, *Beta-,* and *Gamma proteobacteria* and so on (9–12). The process of elemental sulfur generation has been studied mainly through classical biological and chemical methods. The structure and transport of elemental sulfur were usually determined by X-ray near edge absorption spectroscopy (XANES), high-performance liquid chromatography (HPLC) (13–15), and transmission electron microscopy (16, 17). The dynamic changes in metabolites in sulfur metabolism have been measured using chromatography, such as ion chromatography or chemometrics (18, 19). These methods only describe microbial metabolism at a specific point in time, and cannot be used to observe changes on the time scale of individual samples repeatedly. Some of them required destroying cells and preparing samples through complex steps, which might suffer from uneven sampling and contamination, resulting in the difficulty of achieving continuous observations and measurements hardly achieved.

In recent years, Raman spectroscopy has been widely used to observe biological structures and their spatial distribution due to its low cost, rapid, label-free, and nondestructive nature (20–23), making nondestructive *in-situ* continuous observations possible (23). Raman spectroscopy combined with isotope techniques has allowed quantitative labeling, differentiation, and monitoring of growth rates and carbon flow of bacteria (24–26). However, it is complicated due to the need for prior knowledge of metabolic processes and often ignores the spatial distribution patterns of substances. Comparatively, volume quantification using Raman quantitative imaging technology can avoid the above shortcomings, and confocal Raman microscopy (CRM) with higher depth resolution can perfectly link identification, visualization, and quantification under the premise of label-free, nondestructive molecular specificity (23, 27). It has been successfully applied to the study of cells to understand complex mechanisms and interactions (23).

In our previous study, we identified a pathway mediating the formation of elemental sulfur in the deep-sea bacterium Erythrobacter flavus 21-3 through classical biological and chemical methods (19). Based on our previous study, we found that the 21-3 strain was able to utilize the formed elemental sulfur once the nutrient was depleted (28). However, the dynamic process of formation and utilization of elemental sulfur in the 21-3 strain was elusive. Here, based on CRM 3D imaging, we visualized and quantitatively assess bacterial dynamic sulfur metabolism in near real time. Through this method, we detected the growth and sulfur oxidization of *E. flavus* 21-3 *in situ* for over 45 days. The more detailed descriptions of sulfur metabolism of *E. flavus* 21-3 were found to perfectly complement the previous research results.

## RESULTS AND DISCUSSION

### Development of confocal Raman quantitative 3D imaging (CRQI) analysis of bacterial colonies growing on solid medium.

**FIG 1:**
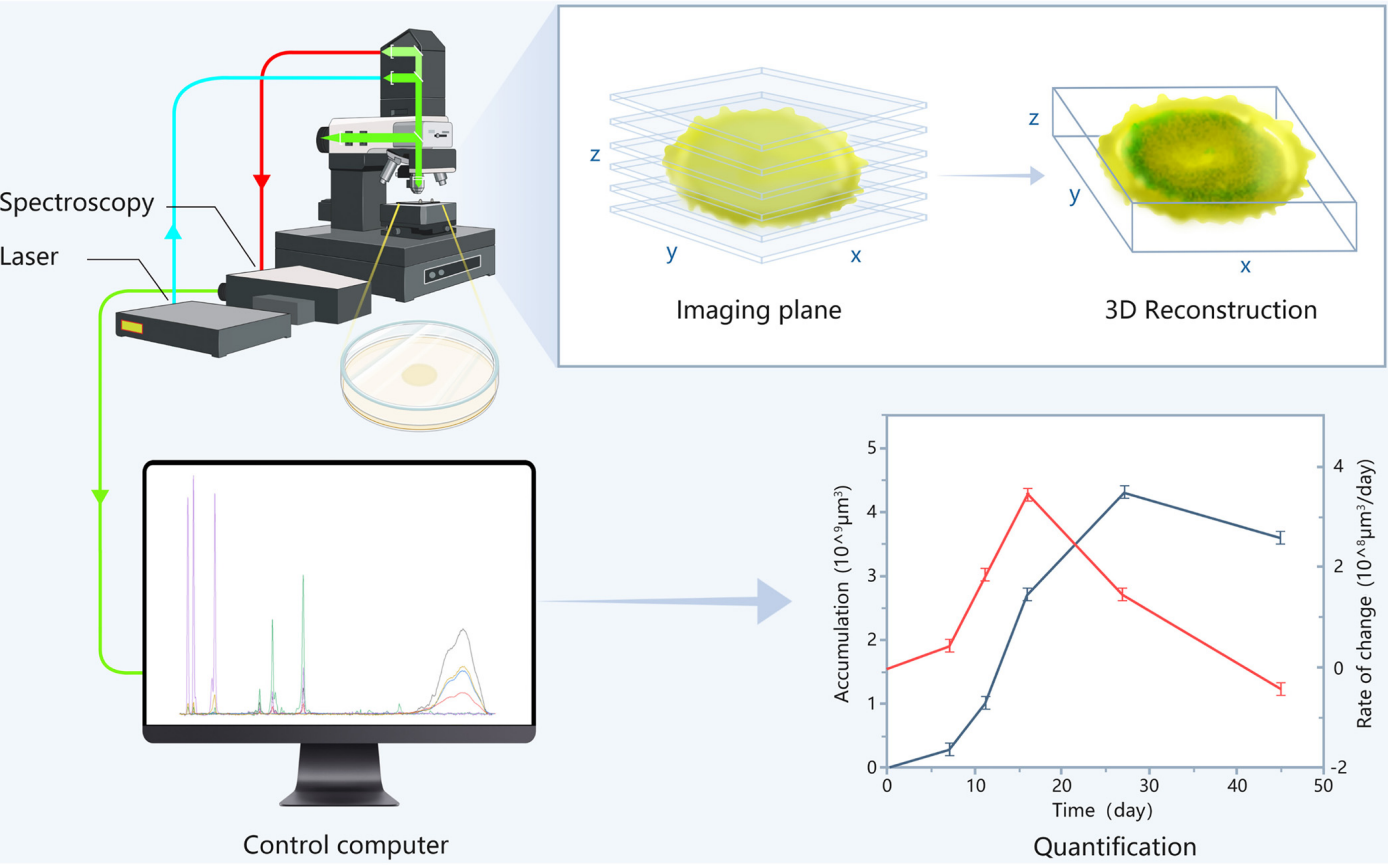
Schematic diagram of 3D quantitative imaging of colonies on solid medium. The laser of the confocal Raman microscope was focused on bacteria grown on the solid medium. Visualization was achieved by 3D scanning, and quantification could be achieved by further data processing.

To better disclose the metabolic process of bacterial colonies, we used a confocal Raman method to quantitatively monitor the growth of bacterial colonies on solid medium with near real-time nondestructive *in-situ* visualization (Fig. 1). First, different characteristic peaks were assigned by spectral comparison and identified as key metabolites based on differences in the Raman peaks. Colonies on the medium were scanned directly with CRM equipped with a ×20 lens objective and a 532 nm laser without any prior pretreatment. According to the characteristic peaks of the metabolite, univariate imaging was performed. Data from each layer were combined to form a three-dimensional (3D) spatial distribution map of the metabolite. Then, separate images of different metabolites were merged to obtain a summarized 3D image of different metabolites. Changes in the metabolic status of the colonies were visualized by near real-time monitoring during the cultivation process. Since the accumulation of biomolecules was associated with each voxel (23), we obtained the cumulative amount and rate of change of each metabolite by calculating its volume in the spatial range. In addition, we took the average spectrum of the 3D imaging, fit the characteristic peaks of metabolites containing the same elements, and analyzed the transformation between metabolites by analyzing the obtained peak area ratios. Finally, we connected quantification with 3D visualization in two ways.

### Preliminary identification of bacterial growth and sulfur metabolism by CRM.

**FIG 2:**
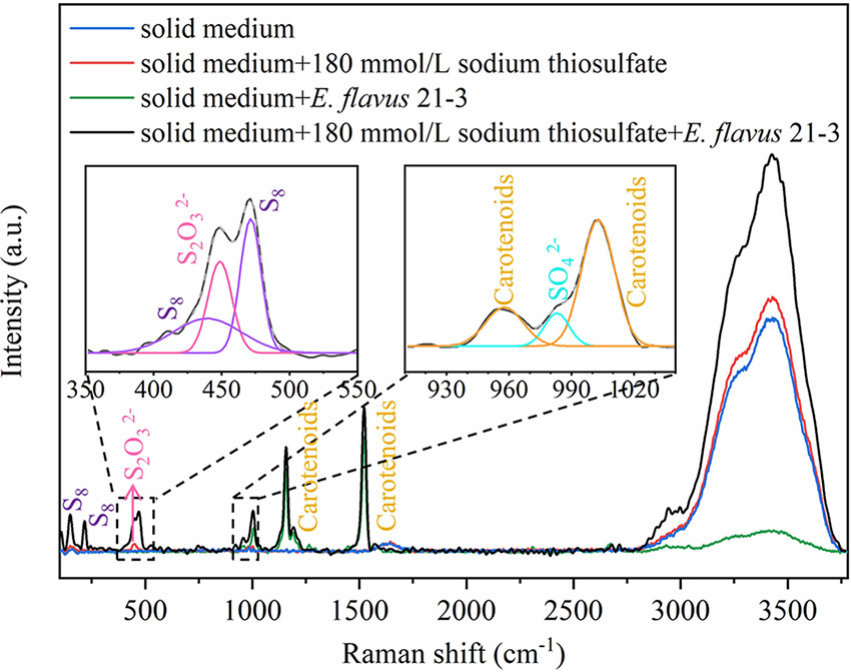
Comparison of Raman spectra from solid medium under different conditions. The blue curve represents the Raman spectra of 2216E solid medium; the red curve represents 2216E solid medium supplemented with 180 mM sodium thiosulfate; the green curve represents *E. flavus* 21-3 cultivated in 2216E solid medium with 180 mM sodium thiosulfate; and the black curve represents *E. flavus* 21-3 cultivated in 2216E solid medium without any thiosulfates. The amplification is the Gaussian fitting of the Raman spectra of the black curve at 350–550 cm^−1^ and 920–1040 cm^−1^. The purple spectrum shows sulfur, the pink spectrum shows thiosulfate, the yellow spectrum shows carotenoids, and the cyan spectrum shows sulfate.

To demonstrate the application potential of this method in the study of bacterial sulfur metabolic processes, we attempted to find a research object. *E. flavus* 21-3 is a sulfur-oxidizing bacterium isolated from cold seep sediments, where microbial mats have been found (29). *E. flavus* 21-3 presented obvious characteristics of sulfur metabolism when it grew on solid medium. Therefore, it is a potentially good research object for testing our system. First, we quantified the rates of division and growth of *E. flavus* 21-3 in the presence and absence of the Raman imaging system using the colony formation unit (CFU) counting method (Fig. S1). It was determined that the 21-3 strain would not be damaged by this method. Then, to evaluate this method, we detected the growth and sulfur metabolism of *E. flavus* 21-3 on solid medium using this method (Fig. 2). Strong Raman peaks appearing at approximately 1,157 cm^−1^ and 1,523 cm^−1^ were found after cultivating bacteria, which represent the C-C and C = C stretching modes of carotenoids (30–32). This result indicated that carotenoids were a characteristic metabolite of *E. flavus* 21-33 and were regarded as a marker of *E. flavus* 21-3 to evaluate its growth. To ensure this, we measured the relationship between the concentration of carotenoids and the biomass of *E. flauvs* 21-3. We found that they are proportional. Therefore, we chose carotenoids for assessing the growth of *E. flavus* 21-3 (Fig. S2).

By comparing the 2216E solid medium with and without sodium thiosulfate, the Raman peak of sodium thiosulfate in the medium was found at approximately 444 cm^−1^ (33). When *E. flavus* 21-3 was cultivated with 180 mM sodium thiosulfate, Raman peaks at 152 cm^−1^, 220 cm^−1^, and 470 cm^−1^ were detected, which correspond to the bending and stretching modes of the 8-fold ring and the vibration of the S-S bond, respectively (1, 34, 35). The Raman peak intensity indicating sulfate at around approximately 980 cm^−1^ was significantly enhanced, indicating that *E. flavus* 21-3 utilized thiosulfate to convert it into elemental sulfur (S_8_) and sulfate (33). Consistent with the previous results, this illustrated the feasibility of using Raman technology to detect bacterial sulfur metabolism (19). The use of CRM is able to quickly determine metabolites, infer pathways, and quickly screen sulfur-producing bacteria. This study provides a basis for visualization and quantitative methods for subsequent *in-situ* research on microorganisms.

### 3D visualization of bacterial growth and metabolism under hyperoxic conditions.

**FIG 3:**
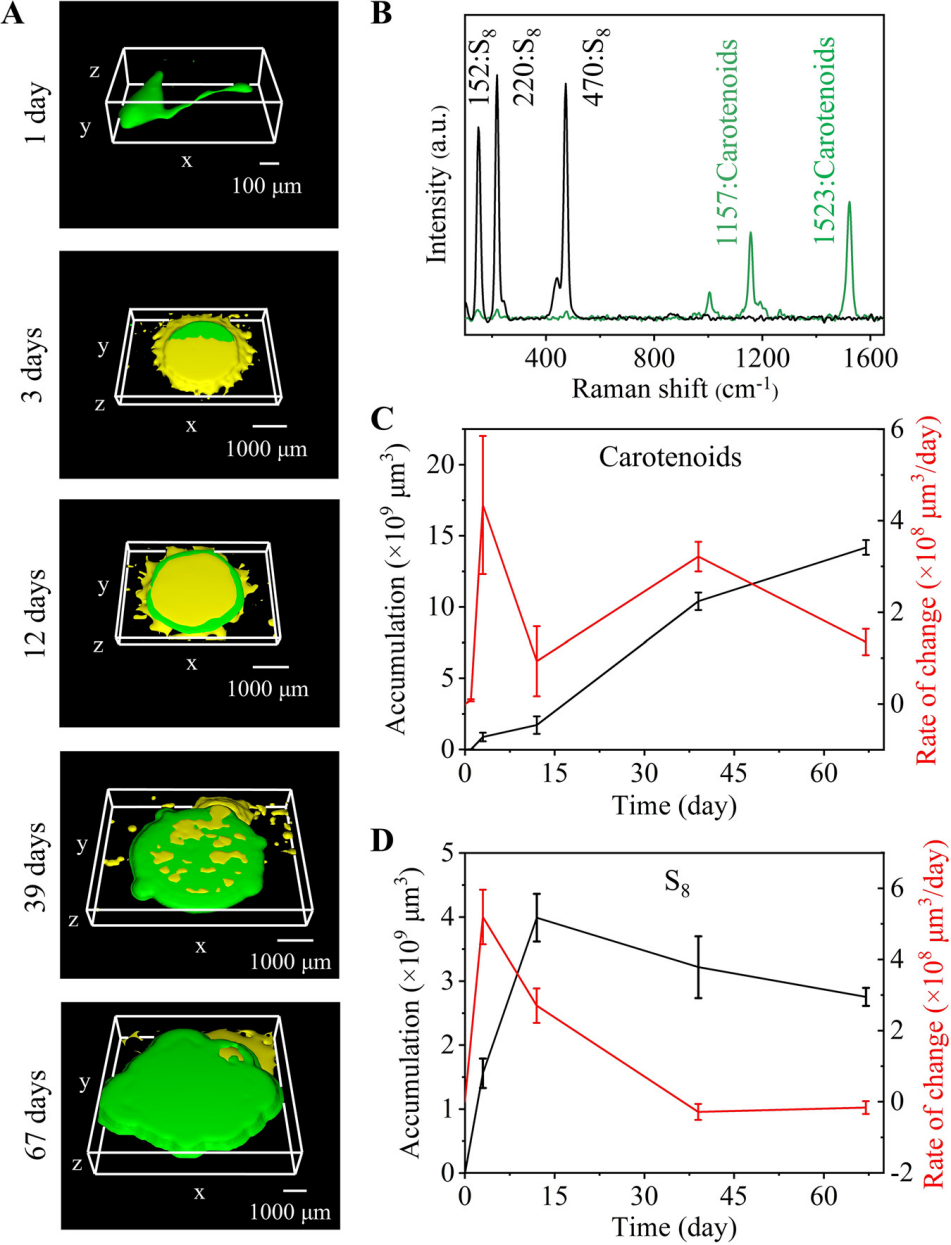
3D imaging analysis of *E. flavus* 21-3. (A) 3D Raman imaging of *E. flavus* 21-3, where yellow represents S_8_ and green represents carotenoids. 3D Raman imaging images of *E. flavus* 21-3 growing under hyperoxic conditions on the 1st day (bounding box size: x × y×z = 725 × 245 × 480 μm), 3rd day (bounding box size: x × y×z = 3855 × 2800 × 565 μm), 12th day (bounding box size: x × y×z = 3960 × 2850 × 655 μm), 39^th^ day (bounding box size: x × y×z = 5645 × 4630 × 770 μm), and 67^th^ day (bounding box size: x × y×z = 6825 × 6825 × 1460 μm). (B) Raman spectrum of *E. flavus* 21-3: black represents the Raman spectrum of the generated S_8_, and green represents the Raman spectrum of the generated carotenoids. (C) Accumulation and rate of change of carotenoids in *E. flavus* 21-3 growing under hyperoxic conditions. (D) Accumulation and rate of change of S_8_ in *E. flavus* 21-3 growing under hyperoxia conditions. Black represents accumulation, and red represents the rate of change.

To verify the capability of near real-time visual monitoring, we first performed univariate imaging of the distinct vibrational modes that could be detected and reconstructed each identified metabolite. The S-S vibration peak at 470 cm^−1^ was used to visualize the content and distribution of S_8_ in the medium (Fig. 3A and B). The carotenoids were visualized using the C = C bond of the polyene chain (1523 cm^−1^) (Fig. 3A and B). The 3D reconstruction of *E. flavus* 21-3 described its growth and sulfur oxidization.

In the early stage (cultivation for approximately 3 days), with bacterial growth, elemental sulfur was continuously secreted to the outside the cell, and a large amount of elemental sulfur accumulated on the surface and edge of the colony (Fig. 3A, Fig. S3A and B). In the middle stage (approximately 12 days), the elemental sulfur was concentrated in the middle of the colony (Fig. 3A and Fig. S3C). Meanwhile, elemental sulfur located at the edge and bottom of the colony disappeared gradually (Fig. 3A, Fig. S3B and C). This result suggested that low concentrations of elemental sulfur located at the edge and the bottom of the colony might start being oxidized or utilized by *E. flavus* 21-3. In the later period (approximately 39 days and longer), the size of the colony gradually increased, indicating that regrowth occurred, and the elemental sulfur on the surface gradually decreased (Fig. 3A, Fig. S3D and E). It was speculated that elemental sulfur or its oxidized products would provide nutrients for the growth of *E. flavus* 21-3. The sulfur metabolic process was consistent with previously reported results (20), indicating that the growth state of *E. flavus* 21-3 might be related to its sulfur metabolism.

Based on the above results, the feasibility and advantages of this method were proved. In this way, visualization and sustainable detection were helpful to research the growth of bacteria, their elemental sulfur metabolism, and the spatial distribution of products in more detail (35). These details could not be investigated using the liquid culture method. However, it was essential to study the metabolism of bacteria growing on solid surfaces, especially in the natural environment. Furthermore, cells would not be damaged when detected. This made it possible to describe the *in-situ* bacterial metabolism in the natural environment.

### Facilitation of the description of detailed bacterial metabolism under hyperoxic conditions through volume quantification.

3D imaging allowed us to visually observe the spatial distribution and changes of *E. flavus* 21-3 and its products by detecting carotenoids and elemental sulfur. To elucidate detailed biological processes, we used a volume quantification scheme based on imaging. The volume occupied by each metabolite in the medium was calculated to quantify the accumulation and the change rate (Fig. 3C, D and Table S1). Under this condition, carotenoids were constantly accumulating within 67 days, indicating that the colonies were always growing and expanding (Fig. 3C). The growth of colonies could be summarized into three stages. The first stage was cultivation for 0 to 3 days. This is the most active stage of growth and production of elemental sulfur (Fig. 3C and 3D). Carotenoids, as a marker of *E. flavus* 21-3 strain, continuously accumulated at the fastest speed among the three stages (Fig. 3C). At the same time, elemental sulfur was also produced and accumulated at the fastest speed among the three stages (Fig. 3D). It was speculated that at this stage, *E. flavus* 21-3 mainly used the nutrients in the medium for growth and metabolism, and continuously converted thiosulfates to elemental sulfur, which was ultimately secreted outside the cell. The second stage was cultivation for 3 to 12 days. The accumulation of carotenoids entered a plateau, and elemental sulfur slowly accumulated at a significantly lower rate (Fig. 3C and 3D). It was speculated that at this stage, the colony had used most of the nutrients in the medium. This resulted in slow bacterial growth and even death. The third stage was cultivation after 12 days. Carotenoids were detected to accumulate again as the concentration of elemental sulfur decreased. This result suggested that *E. flavus* 21-3 could utilize the products oxidized by elemental sulfur or even directly use elemental sulfur for growth at this stage.

These results were consistent with those observed by 3D images but described the whole process of elemental sulfur utilization by *E. flavus* 21-3 in more detail. Bacterial sulfur metabolism plays an important role in the sulfur-utilizing bacteria themselves and the sulfur cycle in their living environment (36). Therefore, the quantitative visualization of bacterial cells to study their specific metabolic processes provided valuable insights into the transformation of substances of microbial colonies in near real-time in *in situ* environments (37).

### Discovery of different bacterial metabolism under hypoxic conditions through 3D visualization and quantification.

In the deep sea, sulfur-oxidizing bacteria in the form of colonies live in a microbial mat. *E. flavus* 21-3 is a sulfur-oxidizing bacterium isolated from cold seep sediments, where microbial mats have been found. The oxygen concentration on the surface of the bacterial mats in a cold seep is much lower than that in a laboratory environment (38). To compare and observe the growth and metabolism of sulfur-oxidizing bacteria in a cold seep under hypoxic conditions, i.e., closer to the *in situ* state, taking *E. flavus* 21-3 as an example, we cultured and completely encapsulated the colony in the middle of the solid medium to form a hypoxic environment, which was monitored by CRQI. The dissolved oxygen concentration was ~3.53 mg/L, which was ~48.6% lower than that in the hyperoxic condition in our study and approaches the dissolved oxygen concentration in the cold seep where *E. flavus* 21-3 was isolated (38).

**FIG 4:**
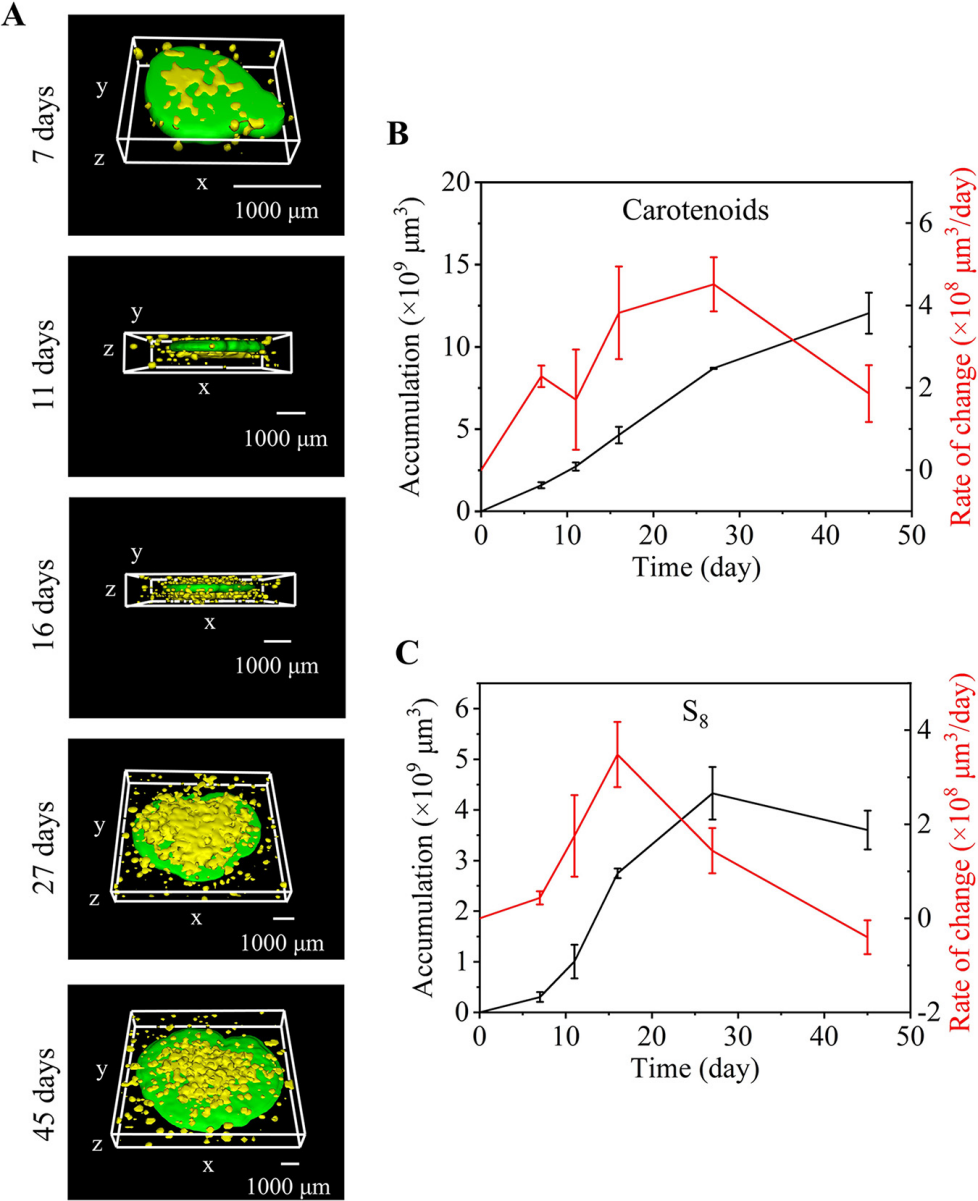
Raman 3D imaging analysis of *E. flavus* 21-3 under hypoxic conditions. (A) 3D Raman image of *E. flavus* 21-3, where yellow represents S_8_ and green represents carotenoids. 3D Raman imaging images of 7th day (bounding box size: x × y×z = 1890 × 1460 × 640 μm), 11th day (bounding box size: x × y×z = 4930 × 4245 × 740 μm), 16th day (bounding box size: x × y×z = 5505 × 4820 × 750 μm), 27^th^ day (bounding box size: x × y×z = 6740 × 6150 × 840 μm), and 45^th^ day (bounding box size: x × y×z = 8395 × 7640 × 1145 μm) of culture under hypoxic conditions. (B) Accumulation and rate of change of carotenoids in *E. flavus* 21-3 under hypoxic conditions. (C) Accumulation and rate of change of S_8_ in *E. flavus* 21-3 under hypoxic conditions.

It was found that under low oxygen conditions, the growth and metabolism of colonies could be summarized into four stages (Table S1). In the first stage (cultivation for 0 to 11 days), compared with the hyperoxic condition, 3D imaging showed that only a small amount of elemental sulfur was detected on the surface of the colony, and carotenoids accumulated at a lower rate (Fig. 4A and B, Fig. S4A and B). It was speculated that under hypoxic conditions, the aerobic sulfur-oxidizing bacteria *E. flavus* 21-3 used the nutrients in the medium to grow slowly in the early stage. Therefore, the production of elemental sulfur was relatively slow, and only a small amount of intracellular elemental sulfur was produced and stored. In the second stage (cultivation for 11 to 16 days), no significant plateau as in the high oxygen conditions was observed (Fig. 4, Fig. S4B and C, Fig S5B and C). Extracellular elemental sulfur began to be generated and accumulated (Fig. 4, Fig. S4B and C), which could be detected on the upper surface and lower surface of the bacterial colony. The amount and generation rate of elemental sulfur increased. It was speculated that this stage was the most active stage for *E. flavus* 21-3 to produce elemental sulfur under hypoxic conditions. The intracellular elemental sulfur increased and was secreted to the outside the cell. In the third stage (cultivation for 16 to 27 days), the middle of the colony began to shrink, and elemental sulfur could only be detected in the depression of the colony (Fig. 4, Fig. S4D and S5D). The rate of elemental sulfur generation declined, although the amount of elemental sulfur continued to increase (Fig. 4C). In addition, the accumulation and generation rate of carotenoids increased (Fig. 4A and 4B). This result suggested that the components that were easily used as nutrients in the medium were exhausted, and the colony began to use part of the elemental sulfur to keep growing. This was also observed under the high oxygen conditions described above. In the fourth stage (cultivation for 27 to 45 days), based on 3D imaging, a large amount of elemental sulfur could still be observed on the surface of the colony (Fig. 4 and Fig. S4E). The accumulation rate of carotenoids continued to increase (Fig. 4B). At the same time, the accumulation of elemental sulfur also began to decline (Fig. 4C). It was speculated that the elemental sulfur generated in the early stage could maintain the late growth of *E. flavus* 21-3.

Through 3D visualization and quantification, we observed different growth and metabolism processes of *E. flavus* 21-3 cultivated under hyperoxic and hypoxic conditions. Compared with the high oxygen condition, with a lower accumulation rate, a lower growth and regrowth rate of the colony was detected under hypoxic conditions (Fig. 3 and 4). It was inferred that sulfur oxidation was one of the important energy sources for *E. flavus* 21-3. In addition, with a high concentration of oxygen, *E. flavus* 21-3 grew again on the surface of extracellular elemental sulfur, and elemental sulfur gradually became undetectable (Fig. 3A and Fig. S3). However, in the later period of hypoxic conditions, elemental sulfur was still exposed on the surface of the colony and was not covered by strains growing later (Fig. 4A and Fig. S4). Due to the possible role of elemental sulfur as an *E. flavus* 21-3 nutrient, we deduced that it might be a smart strategy for *E. flavus* 21-3 to prevent elemental sulfur from being oxidized too quickly when exposed to a high concentration of oxygen.

This comparison showed that CRQI could evaluate the metabolic growth and metabolism of bacteria under different environmental conditions, screen the optimal metabolic conditions of microorganisms, and compare the sulfur production rate of different strains.

### Ratio analysis as a complement to the metabolic quantification of colonies.

The above results showed that the visualization and quantification of carotenoids and elemental sulfur could be achieved using CRQI. However, due to the poor spectral signal-to-noise ratio in solid media, near real-time quantification of metabolites such as sulfate at lower concentrations could not be achieved. Therefore, to solve this problem, we attempted to quantify the conversion process of sulfur by calculating the ratio of related substances. To obtain the change in the sulfate concentration in the solid medium, we took the average spectrum of the total imaging volume of carotenoids and elemental sulfur in 3D space to improve the signal-to-noise ratio to represent the overall situation of the metabolite (Fig. S6). The peak areas of sulfate, thiosulfate, and elemental sulfur were fitted (Fig. 2), and the ratios of elemental sulfur to thiosulfate (*A*_S_8__/*A*_S_2_O__3__^2−^_) and sulfate to thiosulfate (*A*_SO_4_^2−^_/*A*_S_2_O__3__^2−^_) in different periods were calculated to evaluate the conversion of the metabolites.

**FIG 5:**
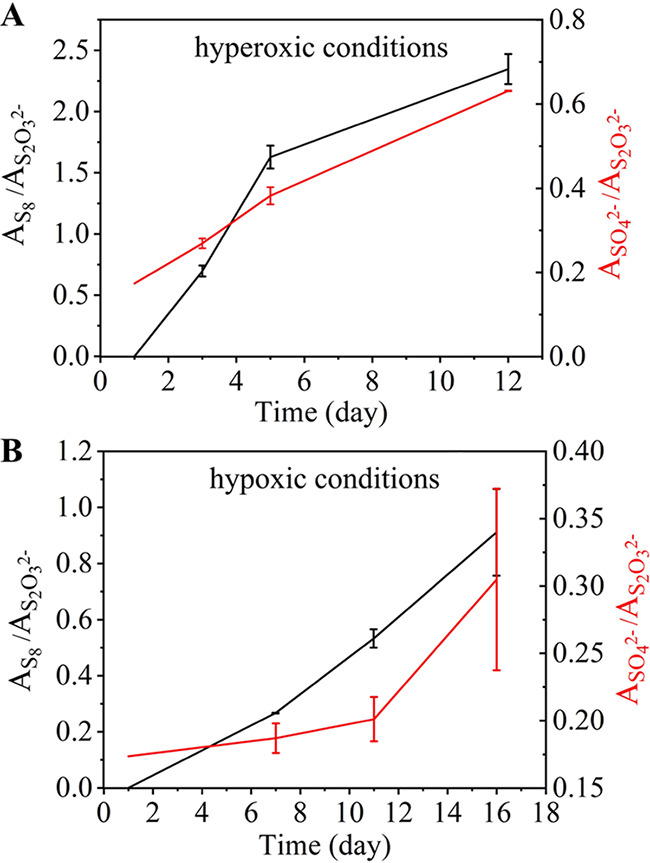
Raman characteristic peak area ratios of species related to sulfur transformation in *E. flavus* 21-3 under different oxygen content conditions. Black represents the ratio of the Raman characteristic peak areas of S_8_ to S_2_O_3_^2−^ (*A*_S_8__/*A*_S_2_O__3__^2−^_), and red represents the ratio of the Raman characteristic peak areas of SO_4_^2−^ to S_2_O_3_^2−^ (*A*_SO_4_^2−^_/*A*_S_2_O__3__^2−^_) under hyperoxic conditions (A) or hypoxic conditions (B).

*A*_S_8__/*A*_S_2_O__3__^2−^_ and *A*_SO_4_^2−^_/*A*_S_2_O__3__^2−^_ showed an increasing trend in both hyperoxic and hypoxic conditions, indicating that *E. flavus* 21-3 could convert thiosulfate to elemental sulfur and sulfate. During the days shown in Fig. 5, *A*_S_8__/*A*_S_2_O__3__^2−^_ and *A*_SO_4_^2−^_/*A*_S_2_O__3__^2−^_ increased first and then slowly under high oxygen conditions, while the opposite trend was observed under low oxygen conditions. The difference in the growth rate indicated that the sulfur metabolism process under these two conditions was different. Comparing the values of the ordinate, it could be found that the value under the high oxygen conditions was larger than that under the low oxygen conditions, indicating that the conversion rate was higher. These phenomena were consistent with our above results, demonstrating that we could use this method to determine the conversion of sulfur. However, the analysis could not be carried out in the later stage because the concentration of sodium thiosulfate decreased, and the method could not continue to provide quantitative evaluation of near real-time detection. It can be seen that both methods have certain limitations, but they can confirm and complement each other.

In summary, combining these two methods, the spatial contribution and dynamic change of metabolites, the conversion of biological processes, and the bacterial response to the environment could be evaluated. The method potentially provides valuable information on biological processes and culture conditions in microbiology. It is expected to be applied in the deep sea for *in situ* studies in the future.

## MATERIALS AND METHODS

### Cultivation medium and growth conditions for *E. flavus* 21-3.

*E. flavus* 21-3 was cultivated in artificial seawater (ASW) marine broth 2216E (1 g yeast extract, 5 g tryptone and 15 g agar in 1 L ASW) at 28°C. One hundred eighty mM sodium thiosulfate was sterilized by a 0.22-μm filter membrane and then added to the autoclaved medium. The ASW contained 24.5 g NaCl, 3.9 g Na_2_SO_4_, 0.7 g KCl, 0.02 g SrCl, 5.0 g MgCl·6H_2_O, 1.1 g CaCl_2_, 0.2 g NaHCO_3_, 0.03 g H_3_BO_4_, and 0.004 g NaF per 1 L of Milli-Q water. The pH was adjusted to between 7.2 and 7.5 using 1 M NaOH. *E. flavus* 21-3 for testing in high-concentration oxygen was cultivated on the surface of ASW marine broth 2216E agar plate. For the low-concentration oxygen test, *E. flavus* 21-3 cultivated on the surface of a 2216E agar plate was covered by the same autoclaved solid medium.

### Raman spectroscopy acquisition and CRM imaging.

Raman spectral collection and 3D Raman imaging were conducted by a confocal Raman micro-spectroscope (alpha 300R, WITec, Ulm, Germany) equipped with a laser with an excitation wavelength of 532 nm. To ensure the consistency of imaging, we placed the colony in the petri dish directly under the lens and kept the same angle for each measurement. Raman spectra were collected using a back-illuminated charge-coupled device (CCD) camera thermoelectrically cooled to –60°C, and an OLYMPUS SLMPlan N 20×/0.25 lens was chosen. A 600 grooves/mm grating (UHTS 300, spectroscopic resolution of 3 cm^−1^) was selected to obtain the spectral range of 0 to 3,000 cm^−1^. When acquiring a single spectrum, the power was 30 mW and the integration time was 0.25 s. To ensure that the detection time was similar for each test, the choice of the step size depended on the range of colony growth. We first determined the approximate range from the xyz direction by line sweeping separately and selected a volume larger than that range for detection to ensure complete detection. The appropriate step size was calculated by combining the volumes.

The general criteria for selecting the parameters were as follows: (i) the sample will not be damaged; (ii) a relatively high-quality spectrum can be obtained after each scan; and (iii) as the integration time of each point is consistent, the corresponding step spacing is adjusted accordingly to ensure that the total time will not be too different.

### Raman imaging processing procedure.

The software WITec Project plus (Control Five 5.2, WITec Company, Ulm, Germany) was applied to Raman data analysis. All Raman images underwent pretreatment, including cosmic ray removal (CRR) and baseline correction (Graph background subtraction). For CRR, we adjusted the CRR parameters “Filter Size” and “Dynamic Factor” to remove cosmic rays and manually delete the remaining parts. For baseline correction, we modified the background subtraction “shape” parameters “Shape Size” and “Noise Factor” to subtract the background, and the same parameters were used for each layer of images. Based on the analysis of components, univariate imaging was conducted by WITec Project plus software for specific vibrational modes, including carotenoid-rich regions (1523 cm^−1^) and S_8_-rich regions (470 cm^−1^). The univariate images of each layer were synthesized and imported into IMAGE J for 3D visualization and volume calculation. The volume calculation is based on the presence or absence of the substance and does not take into account the concentration variation reflected by the difference in the peak intensity. Although the intensity of the Raman peak appears to weaken significantly with increasing depth in the Z-axis direction, we found that the signal of the substance of interest was always detectable in the detection depth range of our present study.

### Spectral processing for ratio analysis to quantify the conversion process of sulfur.

The average spectra of carotenoids and S_8_ were obtained after 3D imaging using the data processing software Project Five 5.2 (Control Five 5.2, WITec Company, Ulm, Germany) to represent the overall colony growth. The spectra were processed using GRAMS/AI 9.3 (Thermo Fisher Scientific, Inc., Waltham, MA, USA) spectroscopic data processing software. After baseline correction, the Gaussian function was used to perform peak fitting to obtain the band position, bandwidth, band height, and area. The ratio of different peak areas of substances related to sulfur metabolism was calculated to obtain the reaction process.

### Data availability.

The Raman spectral data were deposited in the Figshare web site (https://doi.org/10.6084/m9.figshare.21732149.v1).
