# Supplementary material for: Study of Microbial Sulfur Metabolism in a Near Real-Time Pathway through Confocal Raman Quantitative 3D Imaging
Source: Microbiol Spectr. 2023 Feb 21;11(2):e03678-22. doi: 10.1128/spectrum.03678-22 (PMC10101092; doi:10.1128/spectrum.03678-22)
Supplement: Supplemental file 1 — Supplemental material. Download spectrum.03678-22-s0001.pdf, PDF file, 0.6 MB [file spectrum.03678-22-s0001.pdf]

Supplementary Information

Study of microbial sulfur metabolism in a near real-time pathway through confocal Raman quantitative 3D imaging

Wanying He[1,2,3,4#], Ruining Cai[3,4,5,6#], Shichuan Xi[1,2,4], Ziyu Yin[1,2,3,4], Zengfeng Du[1,2,4], Zhendong Luan[1,2,3,4], Chaomin Sun[3,4,5,6*], Xin Zhang[1,2,3,4*]

[1] CAS Key Laboratory of Marine Geology and Environment & Center of Deep Sea Research, Institute of Oceanology, Chinese Academy of Sciences, Qingdao, China

[2]Laboratory for Marine Geology, Pilot Laboratory for Marine Science and Technology, Qingdao, China

[3]College of Earth Science, University of Chinese Academy of Sciences, Beijing, China

[4]Center of Ocean Mega-Science, Chinese Academy of Sciences, Qingdao, China

[5]CAS Key Laboratory of Experimental Marine Biology & Center of Deep Sea Research, Institute of Oceanology, Chinese Academy of Sciences, Qingdao, China

[6]Laboratory for Marine Biology and Biotechnology, Pilot National Laboratory for Marine Science and Technology, Qingdao, China

# These authors contribute equally to this work.

[*] Corresponding authors

Xin Zhang

Tel.: +86 532 82898523; fax: +86 532 82898523.

E-mail address: xzhang@qdio.ac.cn

Chaomin Sun

Tel.: +86 532 82898857; fax: +86 532 82898857.

E-mail address: sunchaomin@qdio.ac.cn

Table S1 Accumulation and rate of change of carotenoids and S_8 in *E. flavus* 21-3 growing under hyperoxic and hypoxic conditions.

Days	Carotenoids		S_8	
	Accumulation (μm^3)	Rate of change (μm^3/day)	Accumulation (μm^3)	Rate of change (μm^3/day)
Hyperoxic conditions				
1	$8.09\times10^{6}\pm2.27\times10^{6}$	$8.09\times10^{6}\pm2.27\times10^{6}$	0	0
3	$8.77\times10^{8}\pm3.02\times10^{8}$	$4.34\times10^{8}\pm1.51\times10^{8}$	$1.56\times10^{9}\pm2.30\times10^{8}$	$5.19\times10^{8}\pm7.65\times10^{7}$
12	$1.72\times10^{9}\pm6.18\times10^{8}$	$9.32\times10^{7}\pm7.64\times10^{7}$	$3.99\times10^{9}\pm3.71\times10^{8}$	$2.70\times10^{8}\pm4.85\times10^{7}$
39	$1.04\times10^{10}\pm6.18\times10^{8}$	$3.22\times10^{8}\pm3.24\times10^{7}$	$3.22\times10^{9}\pm4.82\times10^{8}$	$-2.86\times10^{7}\pm2.25\times10^{7}$
67	$1.42\times10^{10}\pm5.15\times10^{8}$	$1.35\times10^{8}\pm2.87\times10^{7}$	$2.75\times10^{9}\pm1.41\times10^{8}$	$-1.66\times10^{7}\pm1.79\times10^{7}$
Hypoxic conditions				
7	$1.60\times10^{9}\pm1.84\times10^{8}$	$2.28\times10^{8}\pm2.63\times10^{7}$	$3.05\times10^{8}\pm9.76\times10^{7}$	$4.35\times10^{7}\pm1.39\times10^{7}$
11	$2.73\times10^{9}\pm2.51\times10^{8}$	$1.71\times10^{8}\pm1.22\times10^{8}$	$1.01\times10^{9}\pm3.33\times10^{8}$	$1.75\times10^{8}\pm8.67\times10^{7}$
16	$4.64\times10^{9}\pm5.03\times10^{8}$	$3.82\times10^{8}\pm1.12\times10^{8}$	$2.75\times10^{9}\pm9.62\times10^{7}$	$3.48\times10^{8}\pm6.93\times10^{7}$
27	$8.70\times10^{9}\pm4.14\times10^{7}$	$4.51\times10^{8}\pm6.59\times10^{7}$	$4.33\times10^{9}\pm5.20\times10^{8}$	$1.44\times10^{8}\pm4.81\times10^{7}$
45	$1.20\times10^{10}\pm1.25\times10^{9}$	$1.86\times10^{8}\pm6.92\times10^{7}$	$3.60\times10^{9}\pm3.84\times10^{8}$	$-4.03\times10^{7}\pm3.59\times10^{7}$

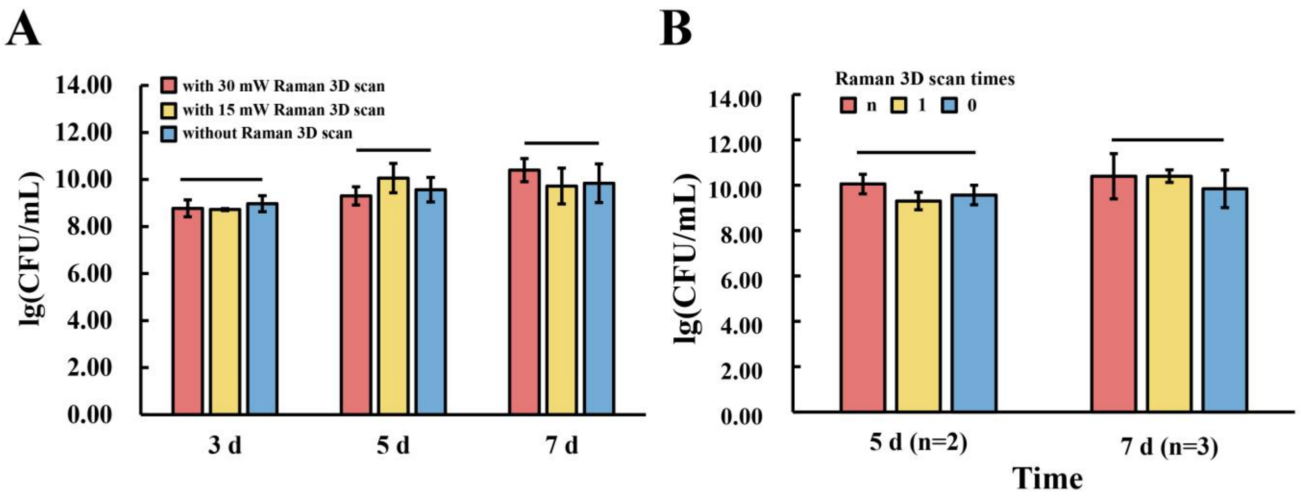

Fig. S1 Measurements of the growth and division of *E. flavus* 21-3 after Raman scans. (A) Compared with the absence of a Raman imaging system, Raman scans with powers of 15 mW and 30 mW showed a nonsignificant influence on the growth and division of *E. flavus* 21-3. (B) Compared with the absence of a Raman imaging system, multiple Raman scans showed a nonsignificant influence on the growth and division of *E. flavus* 21-3.

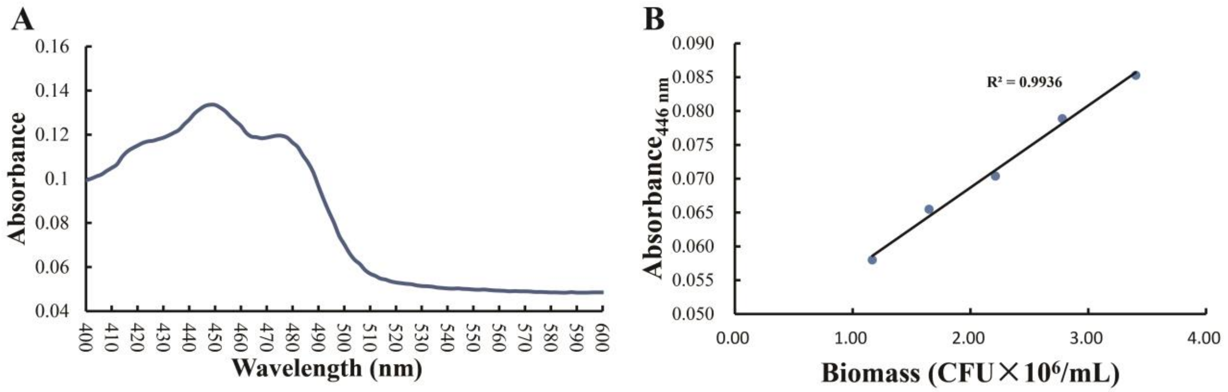

Fig. S2 Characteristics of carotenoids produced by *E. flavus* 21-3. The maximum absorption peak of carotenoids generated by *E. flavus* 21-3 was detected (A). The relationship between biomass and the concentration of carotenoids was detected (B).

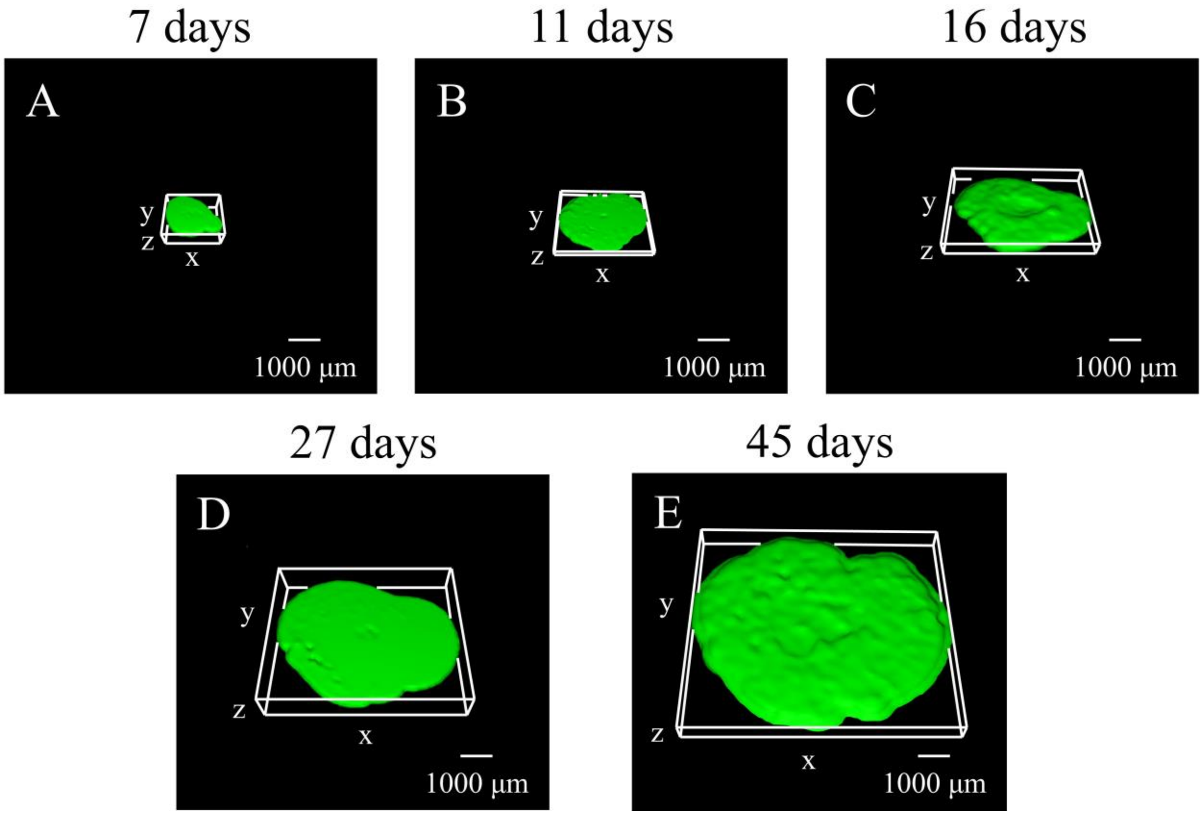

Fig. S5 Raman 3D imaging analysis of carotenoids of *E. flavus* 21-3 growing under hypoxic conditions on the 7th day (bounding box size: $x \times y \times z = 1680 \times 1380 \times 300$ μm) (A), 11th day (bounding box size: $x \times y \times z = 2860 \times 2510 \times 380$ μm) (B), 16th day (bounding box size: $x \times y \times z = 4080 \times 3326 \times 420$ μm) (C), 27th day (bounding box size: $x \times y \times z = 5840 \times 4800 \times 450$ μm) (D) and 45th day (bounding box size: $x \times y \times z = 8000 \times 7100 \times 720$ μm) (E) .

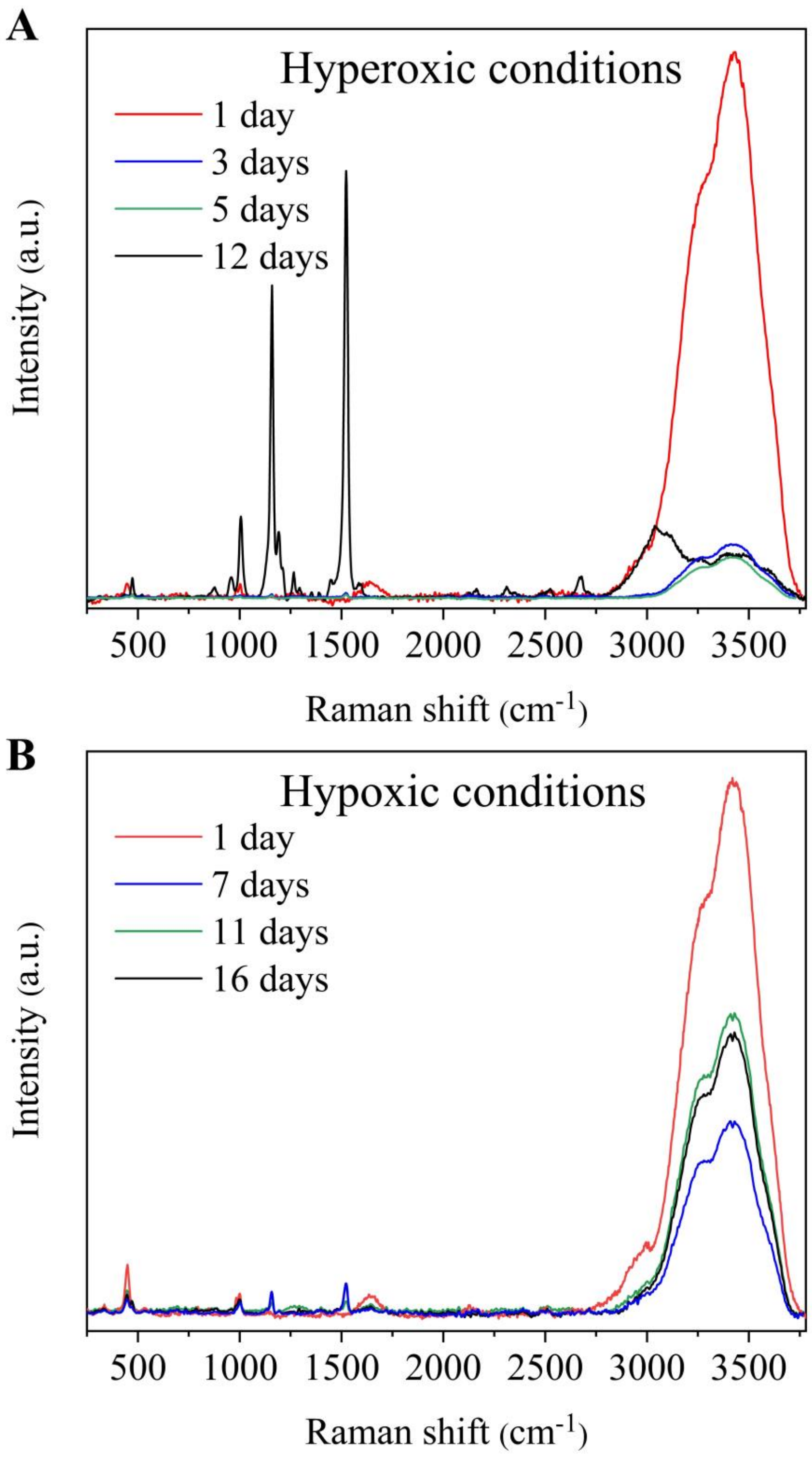

Fig. S6 Quantitative ratio analysis of the original spectra under hyperoxic conditions (A) and hypoxic conditions (B).
