# Supplementary figures and images for: Study of Microbial Sulfur Metabolism in a Near Real-Time Pathway through Confocal Raman Quantitative 3D Imaging
Source: Microbiol Spectr. 2023 Feb 21;11(2):e03678-22. doi: 10.1128/spectrum.03678-22 (PMC10101092; doi:10.1128/spectrum.03678-22)

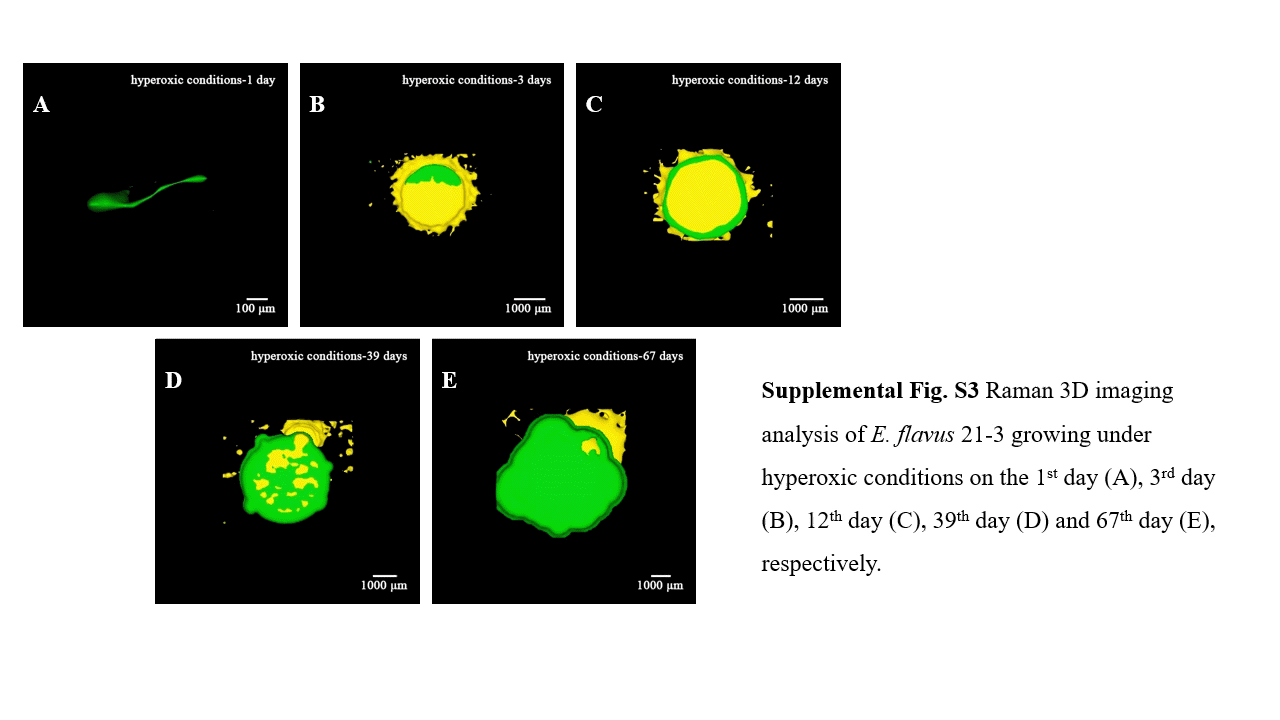

Supplement: Supplemental file 2 — Supplemental material. Download spectrum.03678-22-s0002.gif, GIF file, 4.1 MB [file spectrum.03678-22-s0002.gif]

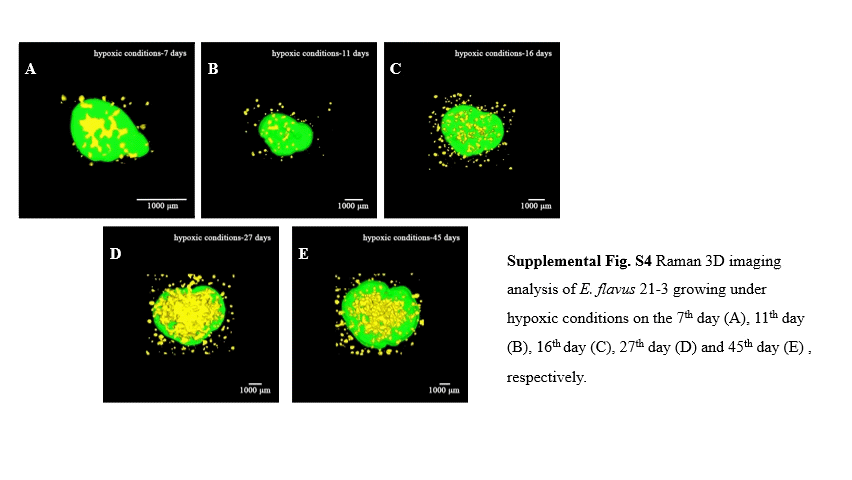

Supplement: Supplemental file 3 — Supplemental material. Download spectrum.03678-22-s0003.gif, GIF file, 2.7 MB [file spectrum.03678-22-s0003.gif]
